# Optimising hyperparameters with a tree structured Parzen estimator to improve diabetes prediction

**DOI:** 10.1038/s41598-025-19295-x

**Published:** 2025-10-10

**Authors:** Raafat M. Munshi, Lammar R. Munshi, Hanen Himdi, Amjad Qashlan, Reema Munshi, Othman Y. Alyahyawy, Mashael M. Khayyat

**Affiliations:** 1 Department of Medical Laboratory Technology (MLT), Faculty of Applied Medical Sciences, King Abdulaziz University, Rabigh, Saudi Arabia; 2 Medicine and Surgery (MBBS), King Abdulaziz University, Jeddah, Saudi Arabia; 3 Department of Computer Science and Artificial Intelligence, College of Computer Science and Engineering, University of Jeddah, Jeddah, Saudi Arabia; 4 Department of Cybersecurity, College of Computer Science and Engineering, University of Jeddah, Jeddah, Saudi Arabia; 5 Pharmacy Practices Department, College of Pharmacy, Umm al-Qura University, Makkah, Saudi Arabia; 6 Department of Information Systems and Technology, College of Computer Science and Engineering, University of Jeddah, Jeddah, Saudi Arabia

**Keywords:** Diabetes, Machine learning, XGBoost, Optuna, Risk prediction, Laboratory parameters, Diagnostic strategies, Classification and taxonomy, Computational models, Data mining, Data processing

## Abstract

Diabetes is a lifelong condition that occurs when the pancreas loses its ability to secrete insulin or experiences a significant reduction in insulin production. Early identification of high-risk patients is crucial for timely interventions and improved outcomes. Traditional clinical risk prediction models rely on regression analysis using clinical, sociodemographic, and anthropometric data; however, they have limitations in terms of accuracy and generalizability. This research proposes a diagnostic strategy leveraging machine learning (ML) techniques, specifically the XGBoost algorithm optimised with Optuna, to enhance high-risk prediction based on laboratory parameters. The study utilises an open-access diabetes dataset incorporating patient demographics, laboratory test results, and clinical outcomes. Data preprocessing, including cleaning, normalisation, and feature extraction, is performed using an Adaptive Tree-Structured Parzen Estimator (ATPE) and XGBoost model. The proposed model outperforms conventional classification models, achieving 83% accuracy, 80% precision, 78% recall, and a 78% F1 score. A comprehensive correlation and confusion matrix evaluation highlights the model’s effectiveness in distinguishing high-risk patients. Findings indicate that integrating machine learning (ML)-based risk classification frameworks with laboratory test-based diagnostic strategies improves predictive accuracy and patient stratification. However, data quality, population diversity, and real-time applicability remain challenges. Future research should explore the integration of real-time data from wearable devices and expand model deployment to other chronic and rare diseases, enhancing adaptability and clinical decision-making.

## Introduction

Diabetes is a lifelong condition that occurs when the pancreas loses its ability to secrete insulin or experiences a significant reduction in insulin production. Consequently, the patient cannot utilise glucose derived from food effectively, leading to increased blood sugar levels^1^. Diabetes can be divided into two types: type 1 and type 2. Type 1 diabetes may arise from a deficiency in insulin production by the pancreas. In contrast, type 2 diabetes can result from a decrease in insulin production or the body’s cells becoming resistant to insulin^2^. Diabetes is associated with many critical complications such as dysfunction, long-term damage, and failure of the kidneys, eyes, heart, and blood vessels^3^. Type 2 diabetes accounts for about 90% of diabetes cases, and it is considered a silent killer^4^; disease indications may not be noticed for many years^5^.

Lifestyle changes and early diagnosis or medical interventions could help prevent type 2 diabetes from occurring in many high-risk individuals^6,7^. Early diagnosis of diabetes is crucial for effective treatment and maintaining blood glucose levels at a normal level to prevent complications from occurring^8^.

Despite their widespread use, current diagnostic tools for diabetes prediction have notable limitations, particularly in accuracy and generalizability. Traditional clinical risk models often rely on regression analysis of clinical, sociodemographic, and anthropometric variables. While these methods provide valuable insights, recent advancements in machine learning offer the potential to significantly improve the accuracy and reliability of disease prediction, diagnosis, and management. In this regard, a model proposed by Belsti, Moran^9^ which analysis of antenatal care records reaches 85% accuracy, 90% precision, 84% F1 score, and 78% recall, surpassing the performance of traditional statistical methods. The majority of outcome prediction models facilitate early intervention for high-risk individual while also enabling cost-effective screening by identifying low-risk individuals, which may reduce the necessity for glucose tolerance tests^10^. AI and ML are being increasingly utilised to process large datasets, uncovering patterns that allow for the rapid identification of high-risk patients and timely interventions. However, ongoing monitoring and frequent updates of diagnostic models are essential to maintain their relevance as patients’ conditions evolve or change^11,12^.

Challenges in laboratory test-based diagnostic strategies include incomplete testing, poor data quality, result variability, and limited applicability across diverse populations. Addressing these issues requires comprehensive measures such as robust data preprocessing, standardised laboratory protocols, and training models on high-quality, diverse datasets to enhance generalisation and reliability. This research aims to develop diagnostic strategies for identifying high-risk patients by utilising a machine learning-based risk classification framework applied to laboratory parameters, focusing on individuals at increased risk of diabetes.

1. Development of multiple innovative AI models: this work introduces several novel machine learning models designed to predict the likelihood of diabetes in patients based on their laboratory test results.
2. Thorough feature importance analysis: the research offers a detailed assessment of the important features impacting model performance, hence improving interpretability and clinical significance.
3. Comprehensive examination of misclassified instances: an in-depth examination of misclassified instances is performed to reveal underlying trends and offer recommendations for potential enhancements in model architecture and predicted accuracy.

## Related work

Current diagnostic tools for diabetes, typically based on clinical regression models, often face challenges in accuracy and generalizability. These limitations have driven researchers to explore advanced data-driven methods, particularly machine learning (ML) algorithms, which have shown great promise in integrating diverse clinical variables and enhancing predictive performance.

Recent studies have explored various ML techniques for the early detection and prediction of diabetes. For example, some efforts included a thorough review and analysis of existing literature on the application of ML algorithms in the early prediction and detection of diabetes to evaluate their clinical utility and limitations^13^ . They conducted a narrative review from 2000 to 2023 using different databases such as PubMed, Scopus, Web of Science and Google Scholar, including 14 studies. Across the studies, the common models include XGBoost, LightGBM, Random Forest, Gradient Boosting, Logistic Regression, Naïve Bayes (Gaussian, Bernoulli), Support Vector Machine (SVM), and Deep Neural Networks (DNN), which could reached an accuracy of 95%. Additionally, they found that XGBoost and Gradient Boosting models often outperformed other methods. However, several studies have demonstrated that machine learning models often outperform traditional logistic regression, although the differences are not always statistically significant^13^.

In 2020, the Author of^14^ compared the performance of eight machine learning models and two conventional logistic regression models in predicting diabetes using routine clinical and biochemical data from early pregnancy. They conclude that a prediction model using either Gradient Boosting Decision Trees (GBDT) or logistic regression can be employed for early diabetes risk assessment, with risk thresholds of 30% (low) and 70% (high). However, machine learning does not clearly outperform traditional regression in their setting^14^. Another study conducted by^15^ compares the performance of machine learning models such as Decision Tree (DT), Random Forest (RF), and XGBoost for early prediction of diabetes using clinical datasets. The Decision Tree model is the most effective among the three for early diabetes prediction on the given dataset, achieving an accuracy of 84%. However, the study emphasizes the need for larger, multi-center datasets for broader generalizability. Also, it needs to perform advanced imputation or feature engineering, which could improve the performance^15^. To enhance the predictions of diabetes, the author of^16^ integrates clinical and biochemical data to develop diabetes Predictor, a machine learning-based tool for early prediction and personalized treatment of diabetes. The diabetes Predictor, powered by Random Forest, could achieve a high accuracy (AUC = 96.7%) when combining clinical and biochemical data, making it a promising tool for early diabetes risk assessment and personalized treatment^16^.

Other recent studies have focused on deep learning^17–19^ and transformer-based models^20,21^, demonstrating their potential to enhance traditional ML techniques in detecting diabetes. These models can capture complex temporal dependencies and multimodal relationships within health data, leading to a more precise identification of high-risk individuals and supporting personalized intervention strategies. Despite these advances, challenges remain to ensure clinical applicability and generalization of these models in diverse populations.

To summerize , these studies on diabetes prediction (e.g.,^13–16^) have consistently demonstrated a promising potential of machine learning (ML) models in forecasting both Gestational Diabetes Mellitus (GDM) and type 2 diabetes risks. In particular, tree-based ensemble methods–including Gradient Boosting, Random Forest, and XGBoost–have frequently outperformed traditional approaches, with reported accuracies ranging from 74% in a large-scale Chinese cohort study to 96.7% in the diabetes Predictor study, which incorporated both clinical and biochemical variables. While some research, such as^14^ and the China cohort study^15^, reported that logistic regression models yielded performance comparable to ML-based alternatives, the diabetes Predictor model^16^ achieved superior outcomes, largely attributed to its use of a broader feature set and a web-based deployment strategy. These findings collectively highlight the added value of integrating heterogeneous clinical and biochemical data in enhancing early detection capabilities and enabling more tailored risk stratification strategies

In this study, we focus on developing a machine learning-based risk classification framework using laboratory parameters for the early detection of diabetes. We employed an XGBoost model, optimized with the Optuna hyperparameter tuning framework, to enhance performance and generalizability, which was limitedly explored in previous studies.

## Methodology

**Fig. 1:**
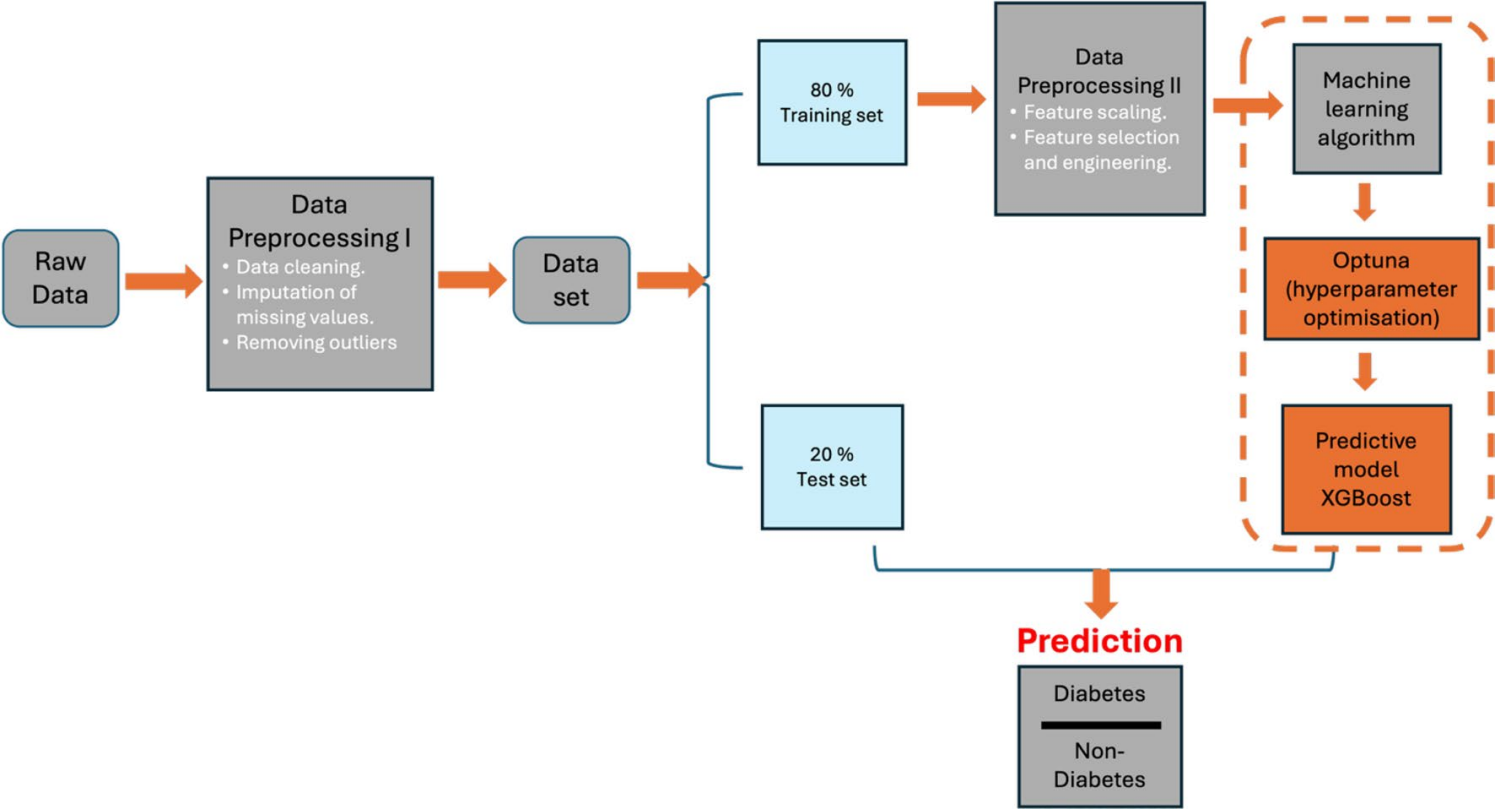
Research design for diagnostic strategies for identifying high-risk patients. The raw dataset undergoes initial preprocessing (missing value imputation, outlier removal), followed by splitting into training and test sets. Feature scaling and selection are then applied to the training data, which is fed into a machine learning model (XGBoost) with hyperparameter tuning via Optuna.

The dataset is analysed using visual methods to explore key features, which is usually called Exploratory data analysis^22^. This approach aims to explore the dataset to identify underlying patterns and relationships between clinical parameters. Figure 1 illustrates the research design, which incorporates patient records, laboratory test results, demographic details, and clinical outcomes. The data is cleaned and normalised to ensure quality before preprocessing. Relevant features are extracted from the preprocessed data using XGBoost, optimised by Optuna. This method is employed to classify patients at high risk of diabetes based on laboratory parameters.

### Data source

The dataset was downloaded from the open-access Kaggle website (https://www.kaggle.com/datasets/mathchi/diabetes-data-set).

The dataset included in this research was acquired from the National Institute of Diabetes and Digestive and Kidney Diseases. It has been meticulously selected to facilitate the construction of predictive models for diabetes diagnosis, which are based on a collection of clinical and physiological measurements.

The demographic data attributes are as follows: Population: Women of Pima Indian heritage (Indigenous group in the southwestern United States, primarily in Arizona), Gender: Female only, Age Range: 21 years and older, Sample Size: 768 individuals, Geographic Origin: United States–Phoenix, Arizona area, and Ethnicity: Pima Indian (Native American)To ensure consistency and relevance for diabetes research, severe selection criteria were used to distinguish this cohort from the broader population. The dataset includes a number of features such as Pregnancies: The total count of the patient’s pregnancies, Glucose: Plasma glucose level assessed two hours post-oral glucose tolerance test, Blood Pressure: Diastolic blood pressure (mm Hg), Skin Thickness: Triceps skinfold measurement (mm), Insulin: Serum insulin concentration at two hours (mu U/ml), BMI: Body Mass Index, determined by dividing weight in kilograms by the square of height in meters, DiabetesPedigreeFunction: A function that evaluates the probability of diabetes based on familial history, Age: The patient’s age in years, and Outcome: A binary categorical variable denoting 268 patients as diabetic (1) and 500 patients as no_diabetic (0).

### Data preprocessing

The first phase of data preprocessing is the most critical step in building diagnostic strategies that identify high-risk patients, which is cleaning the data. Human mistakes could result in missing data, equipment malfunction, and inconsistent or absent data being stored. The approach imputes data values to produce a reliable machine-learning model for predicting patients at risk based on laboratory parameters.

For glucose, blood pressure and IBM mode were employed to impute missing data, whereas for insulin and skin thickness, machine learning-based predictions (random forest model) were utilised for the same purpose. This is followed by removing outliers using the $25{\text {th}}$ and $75{\text {th}}$ percentiles based on the interquartile range method.

In the second phase of dataset preprocessing (feature engineering), new features were created, such as the Age-BMI interaction (multiplying age by BMI), glucose-insulin interaction, squaring BMI, and calculating the sum of skin thickness and insulin, along with the glucose-insulin ratio, among others. The range of features was adjusted to ensure they shared the same scale through standardisation (z-score scaling). This was followed by splitting the dataset into 80% for the training set and 20% for the testing set. To provide a more reliable estimate of the outcome, a simple repeated random sub-sampling validation (Shuffle Split) was used.

### Models employed

#### Machine learning models

To develop an effective predictive model for diabetes diagnosis, several machine learning algorithms were applied, each presenting unique benefits in managing clinical data. The chosen models comprise both linear and non-linear classifiers, selected for their shown efficacy in medical data consideration. The architecture and guiding ideas of each model utilised in this investigation are summarised in this section.

Logistic regression A statistical analytical method for determining the outcome of a dataset. This method has become popular in fields such as biology, economics, and medicine due to its simplicity in interpreting numerical data^23^.

Decision tree A supervised machine learning method in which data is divided based on specific parameters. A smaller cluster of data is produced from the original data by applying the concept of nodes and leaves to split the large data into smaller groups^24^.

Random forest A prediction algorithm that combines decision trees, bagging methods, combined regression, classification techniques, and numerous generated decision trees to obtain accurate results^25^.

Support vector machine (SVM) SVM: one of the most commonly used machine learning classification methods. In the linear method of SVM, the data is divided into two decision lines to create a hyperplane that separates classes from one another. In the non-linear method, the kernel is used to enhance the classification accuracy^26,27^.

K-nearest neighbours It is a machine-learning method for classification. It searches for the nearest neighbours in the data^28^ during the estimation process.

#### Developed model (XGBoost with Optuna)

Extreme Gradient Boosting (XGBoost) is an advanced machine-learning algorithm derived from random forests and decision trees. It is efficient, high-performing, and fast. However, it often struggles with accuracy when dealing with complex datasets^29^. As shown in Eq. (1), the initial prediction was set to zero, and each tree was added to reduce the errors.

1 $$\begin{aligned} \hat{y_i} = \sum _{k=1}^{K} f_k(x_i) \end{aligned}$$

Where $\hat{y_i}$ represents the predicted value for (*i*) data point, *K* is the number of trees and $f_k(x_i)$ is the prediction of the *k*th tree of the *i*th data point.

XGBoost consists of the loss function that measures how the model fits the data and the regularisation term, simplifying complex trees as shown in Eq. (2).

2 $$\begin{aligned} obj(\theta ) = \sum _{i}^{n} l(y_i, \hat{y}_i) + \sum _{k=1}^{K} \Omega (f_k) \end{aligned}$$

Where $l(y_i, \hat{y}_i)$ calculates the difference between the actual value and the predicted value. $\Omega (f_k)$ represents the regularisation term that discourages overly complex trees.

The model optimises iteratively and starts with an initial prediction $\hat{y}^{(0)} = 0$, and then adds a new tree to improve the model. The updated predictions added the tree, which can be presented by the following Eq. (3).

3 $$\begin{aligned} \hat{y}_i^{(t)} = \hat{y}_i^{(t-1)} + f_t(x_i) \end{aligned}$$

Where $\hat{y}_i^{(t-1)}$ the prediction from the previous iteration and $f_t(x_i)$ is the prediction.

The regularisation term simplifies complex trees by penalising the number of leaves in the tree and the size of the leaf, as shown in Eq. (4).

4 $$\begin{aligned} \Omega (f_t) = \gamma T + \frac{1}{2} \lambda \sum _{j=1}^{T} w_j^2 \end{aligned}$$

Where *T* is the number of leaves in the tree, $\gamma$ is a regularisation parameter that controls the complexity of the tree, and $\lambda$ is a parameter that penalises the squared weight of the leaves $w_j$.

When deciding how to split the nodes in the tree that calculated the information gain for every possible split, as shown in Eq. (5):

5 $$\begin{aligned} \text {Gain} = \frac{1}{2} \left[ \frac{G_L^2}{H_L + \lambda } + \frac{G_R^2}{H_R + \lambda } - \frac{(G_L + G_R)^2}{H_L + H_R + \lambda } \right] - \gamma \end{aligned}$$

$G_L$ and $G_R$ are the sums of gradients in the left and right child nodes, and $H_L$ and $H_R$ are the sums of Hessians in the left and right child nodes.

XGBoost selected the split that resulted in the most significant gain for every possible split at each node, effectively reducing errors and improving the model’s performance.

Most machine learning algorithms depend on initial conditions (hyperparameters) that affect model performance. However, defining an optimal setting of hyperparameters for a model can sometimes be challenging. Hyperparameter tuning or optimisation requires manual or automated searches to find optimal values, which, depending on dataset complexity, can be expensive and time-consuming. This model was further optimized with Optuna, an open-source optimisation software designed using the define-by-run principle. It allows users to construct the parameter search space dynamically. In addition, it enables efficient implementation of both searching and pruning strategies and provides an easy-to-setup versatile architecture^30^. The former frameworks stand out when addressing hyperparameter optimisation problems in machine learning^31^. Optuna is an open-source hyperparameter optimisation framework for automated machine learning (AutoML). It is compatible with various machine learning libraries, including Scikit-learn, TensorFlow, PyTorch, and XGBoost^32^. According to Akiba, Sano^30^, Optuna provides artistic optimisation algorithms that are used to minimising/maximising an objective function, as shown in Eq. (6), which takes a set of hyperparameters ($\lambda _s$ and $\lambda _c$) via Bayesian optimisation^33^. The pruning function terminates the training evaluation process whenever it detects inadequate training performance and below a specific metric^31^.

6 $$\begin{aligned} \lambda ^* = \arg \min _{\lambda \in \Lambda } \Phi _{\lambda _s} \sum _{c=1}^{n} \mathscr {L}_c\left( \mathscr {A}(\mathscr {D}_c^{train}, \lambda _c), \mathscr {D}_c^{valid} \right) \end{aligned}$$

Optuna is an adaptive approach that utilises a Tree-structured Parzen Estimator (TPE) incorporated into the hyperparameter optimisation algorithm^34^. In Bayesian optimisation, minimisation/maximisation occurs for an unknown objective function *f*(*x*) over a vector *x*, by iteratively choosing *x* and monitoring noisy objective value $y = f(x) + \varepsilon$^33^. The TPE algorithm uses Parzenestimators, a statistical model for density estimation (Kernel density estimator), which is used to select the hyperparameter configuration most likely to be reasonable and least likely to be bad. The update in Optuna enhances TPE to account for hyperparameter dependencies during optimisation. This improvement boosts optimisation performance and enables more efficient hyperparameter tuning.

The Gradient Boosting Classifier was chosen and optimized using the Optuna framework to improve model performance and achieve optimal generalization. The objective function for Optuna was to minimize the prediction error of the model, calculated as one minus the accuracy on the test set. A total of 50 trials were carried out to investigate the specified parameter space and determine the optimal configuration. The final Gradient Boosting model was trained using the optimal parameters and subsequently evaluated on the test set.

Table 1 displays a summary of the parameters with their descriptions adjusted for each compiled model. Note that the ML models compiled used the default parameters for Scikit-learn’s class.

**Table 1:** Parameters in algorithms.

Model	Parameters / description
XGBoost with Optuna	n_estimators: the count of boosting stages, which can vary from 50 to 250 in increments of 50 Learning rate: the parameter that determines the magnitude of weight updates, with a range of 0.01 to 0.2 max_depth: the maximum allowable depth for individual decision trees, which can range from 3 to 7 early stopping: parameters validation_fraction= 0.1 and n_iter_no_change=5
KNN	n_neighbors=5, indicating that classification was based on the majority class among the 5 nearest neighbors
Logistic regression	Regularization norm to be applied (L2 penalty) Regularization parameter C = 1.0, which balances margin maximization and classification error
Decision tree	Criterion: is a function to measure the quality of spilt: set to ‘gini’ Max_depth = none, allowing each tree to grow until all leaves are pure or contain fewer than the minimum required samples
Random forest	n_estimators=100, specifying the use of 100 decision trees Max_depth=none, allowing each tree to grow until all leaves are pure or contain fewer than the minimum required samples
SVC	Radial basis function (RBF) kernel Regularization parameter C = 1.0, which balances margin maximization and classification error

### Correlation matrix

**Fig. 2:**
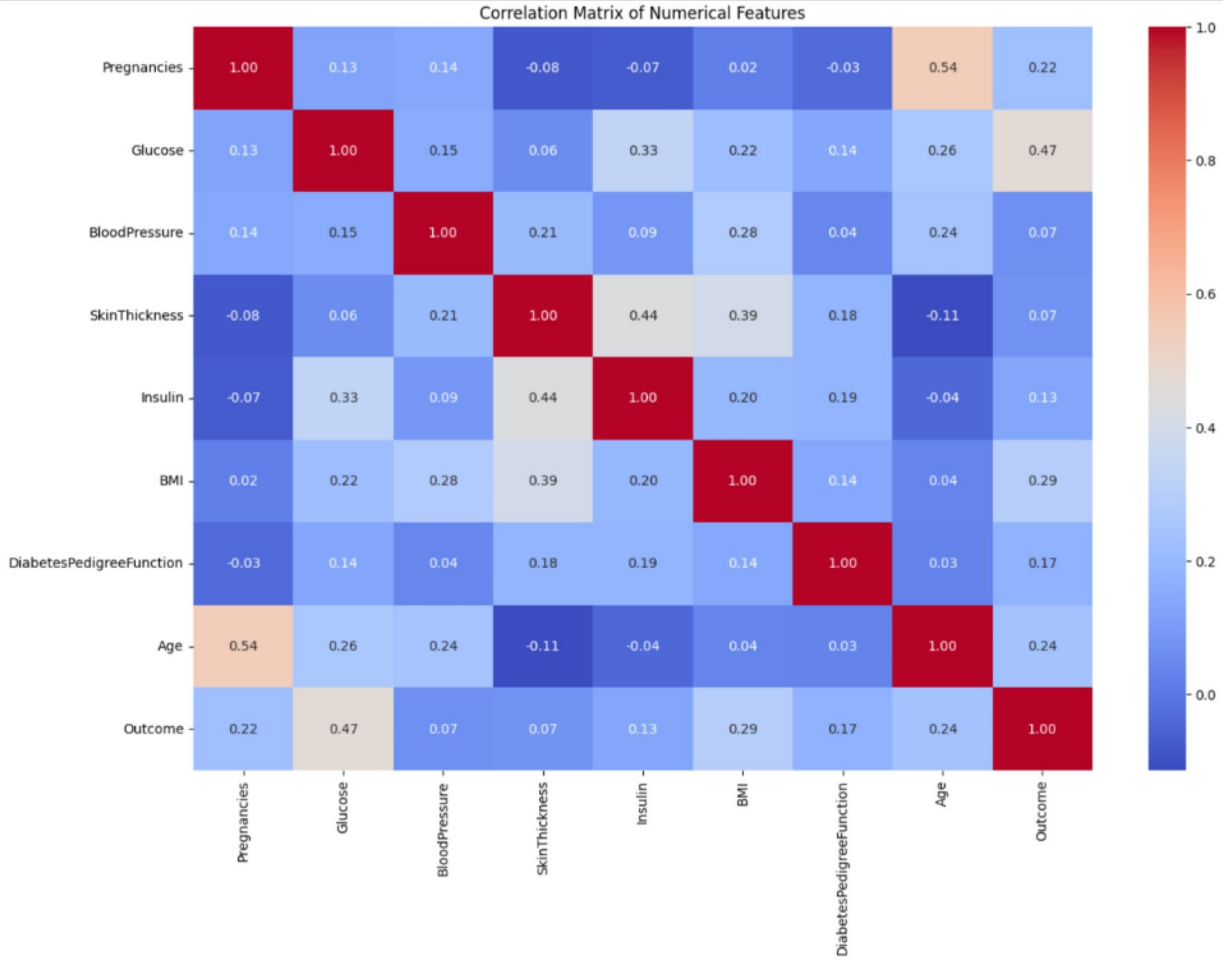
Correlation matrix for diagnostic strategies.

Figure 2 shows the correlation matrix of the linear relationship between clinical and demographic variables, with values ranging from 1 to -1. Most variables exhibit weak correlations (close to 0), indicating minimal linear dependence between the values. Strong self-correlation is observed along each variable’s diagonal (value = 1). Notably, all variables show a low correlation below 0.5, such as diabetes pedigree function, insulin, blood pressure, and skin thickness.

## Experimental setup

The research is based on high-risk patient diagnostic strategies using Python 3.10, supported by Optuna 4.2.1 and XGBoost 2.1.4 methods that are compared with other existing methods for diagnosis^35^. The dataset was initially divided into training and testing subsets utilizing an 80/20 ratio to maintain data integrity and facilitate a fair evaluation process throughout all the developed models.

The model’s accuracy is based on correctly identifying high-risk patients. So, it reflects the percentage of correctly classified high-risk patients, including both true positives (*TP*) and true negatives (*TN*), in all diagnostic cases. It assesses the diagnostic model’s overall reliability and effectiveness in predicting patient risk. On the other hand, precision is calculated as the proportion of *TP* to the total number of *TP* and false positives (*FP*).

Recall, or sensitivity, measures the model’s effectiveness in detecting all actual high-risk patients. It is calculated as the ratio of true positives (*TP*) to the total of false negatives (*FN*) and true positives (*TP*). The *F*1-score balances precision and recall by computing their harmonic mean. This metric is especially useful in scenarios with an imbalance in the distribution of high-risk patient cases.

Performance was evaluated using four standard metrics: accuracy, precision, recall, and *F*1-score, all represented as percentages. The metrics are based on *TP*, *TN*, *FP*, and *FN* stand for True Positive, True Negative, False Positive, and False Negative, respectively. The equation for each evaluation metric is described below:

$$\begin{aligned} \text {Accuracy} = \frac{TP + TN}{TP + TN + FP + FN}, \quad \text {Precision}~(P) = \frac{TP}{TP + TN}, \quad \text {Recall}~(R) = \frac{TP}{TP + FN}, \quad F\text {-Score}~(F) = 2 \left( \frac{P \cdot R}{P + R} \right) \end{aligned}$$

## Results and discussion

Table 2 presents the performance of various machine learning models evaluated for diabetes prediction. Traditional models such as K-Nearest Neighbors (KNN), Logistic Regression, and Decision Trees demonstrated lower classification performance, with accuracies of 70%, 71%, and 69%, respectively. Among them, Logistic Regression performed marginally better.

Support Vector Machine (SVM) and Random Forest showed improved results, achieving accuracies of 75% and 73%, respectively. The SVM model also yielded strong recall (72%) and F1 score (72%), indicating balanced sensitivity and precision. Furthermore, XGBoost models attained a similar performance with an accuracy of 75%, signifying consistent categorization of both diabetic and non-diabetic patients.

**Fig. 3:**
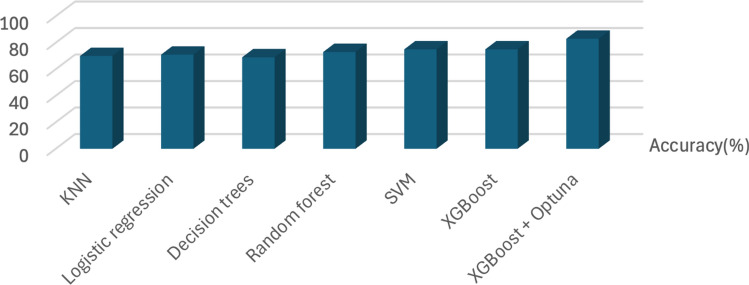
Accuracy performance in diagnostic strategies.

However, when XGBoost was optimized with Optuna, the model achieved the best performance. It surpassed all other models, achieving an accuracy of 83%, precision of 80%, recall of 78%, and an F1 score of 78%. This demonstrates the impact of hyperparameter tuning on enhancing predictive performance in clinical classification tasks. The results confirm that optimization techniques such as Optuna can significantly improve model robustness and accuracy in real-world applications, as summarised in Table 2. Additionally, Fig. 3 shows a bar chart illustrating the accuracy (%) of the various classification models, highlighting the enhanced performance of XGBoost + Optuna (proposed model).

**Table 2:** Summary of model performance.

Methods	Accuracy (%)	Precision (%)	Recall (%)	F1 score (%)
KNN	70	67	66	66
Logistic regression	71	68	67	67
Decision trees	69	66	63	63
Random forest	73	71	70	70
SVM	75	73	72	72
XGBoost	75	72	71	72
XGBoost + Optuna (proposed)	83	80	78	78

The results attained in this study employing the XGBoost model tuned using Optuna line up with those published in previous clinical prediction tasks. Previous research on detecting venous thromboembolism (VTE) implementing ensemble-based models, such as XGBoost and Random Forest, displayed high predictive accuracy and robustness in handling unbalanced clinical datasets^35^. Similar to this study, the research highlights the importance of combining clinical variables with advanced model optimization for effective early detection. Moreover, previous research (e.g.,^13,14,16^) has shown that Gradient Boosting and XGBoost models consistently outperform traditional classifiers like Logistic Regression. Our data support these conclusions, with our proposed model outperforming other models in our baseline comparison, including SVM and Random Forest, with an F1 score of 78% and an accuracy of 83%. Moreover, the proposed model in this study surpassed several baseline algorithms, including Support Vector Machines (SVM) and Random Forest, achieving an F1 score of 78% and an overall accuracy of 83%. These outcomes further validate the efficacy of our modeling approach and underscore the relevance of using feature-rich datasets in improving predictive performance for diabetes risk assessment.

The constancy of performance across varied medical domains (e.g., diabetes, thromboembolic illnesses) demonstrates the generalizability and versatility of ensemble learning approaches, especially when combined with hyperparameter optimization frameworks such as Optuna. It supports the case for using such tailored machine learning models in a variety of clinical prediction tasks, where early and accurate detection might have significant impacts on patient outcomes.

### Confusion matrix

The confusion matrix is a tabular representation utilized to assess the accuracy of classification methods by comparing true with predicted outcomes. It is a widely used approach in machine learning to evaluate a model’s efficacy in differentiating between classes^36^. In medical diagnosis, it aids in measuring true positives (accurately detected cases), true negatives (properly eliminated cases), false positives, and false negatives.

**Fig. 4:**
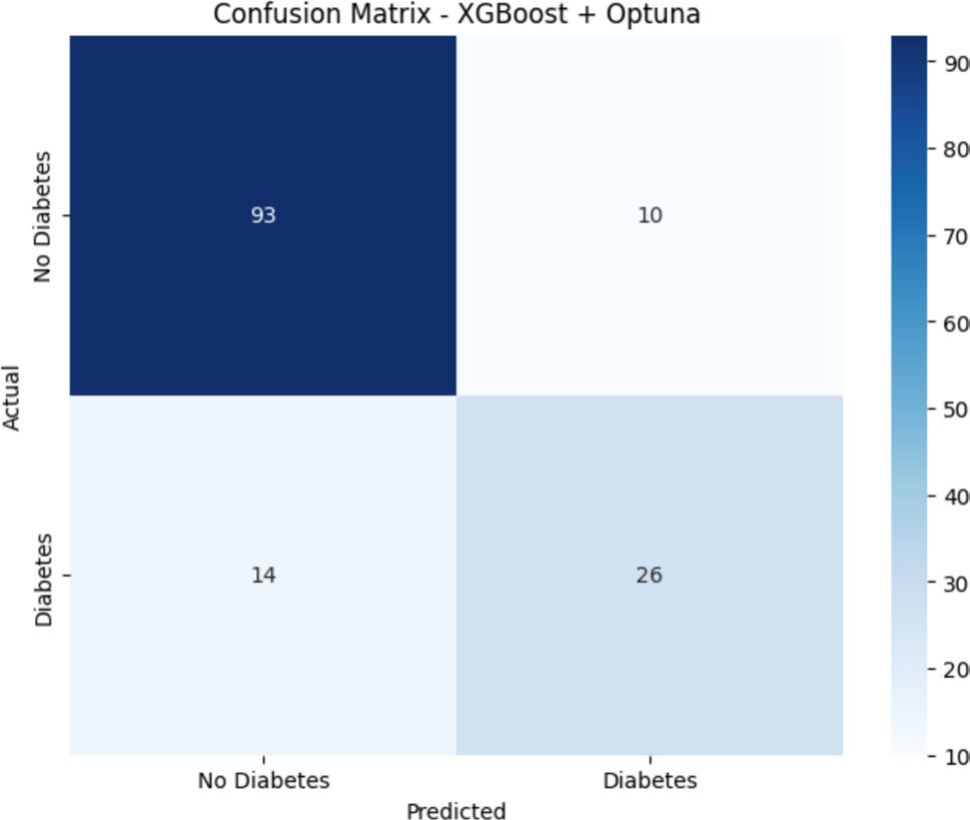
Confusion matrix for the proposed binary classifier model.

As shown in Fig. 4, the confusion matrix for the proposed XGBoost model optimized using Optuna shows a robust and balanced diagnostic ability, with 93 true negatives and 26 true positives, as well as only 14 and 10 false negatives, respectively. This demonstrates the ability of the model to reduce errors, which is a crucial aspect in medical diagnosis, whereby misclassification may result in postponed medical care or inappropriate treatments. A low false negative rate is essential, as accurately diagnosing diabetes patients ensures immediate medical intervention and reduces the risk of complications. Although other machine learning models, including SVM, Random Forest, and Logistic Regression, achieved competitive results, the enhanced XGBoost model surpassed them in every metric. These findings indicate the need to combine ensemble approaches with hyperparameter optimisation to provide precise, dependable, and clinically significant diagnostic tools that facilitate early intervention and improve preventive healthcare measures.

### Features importance

**Fig. 5:**
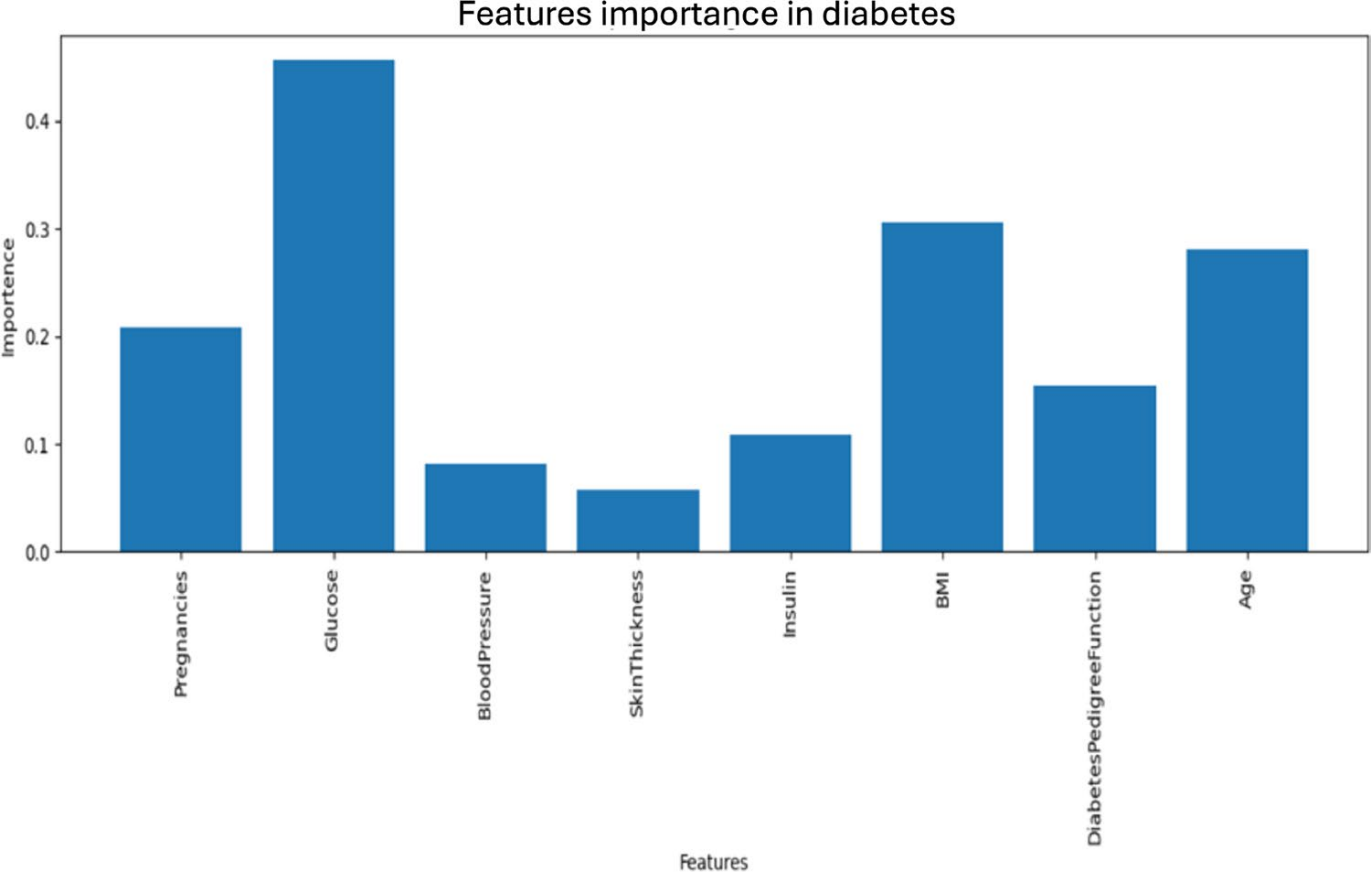
Features importance for diabetes risk satisfaction.

Figure 5 visualises the importance of various features in predicting gestational diabetes risk. Key features, such as glucose, age, number of pregnancies, diabetes pedigree function, and insulin, even when below 0.5, have the highest importance, indicating a strong contribution to the model’s prediction. In contrast, other factors show comparatively lower significance. These insights help prioritise critical features in gestational diabetes risk assessment models.

Ultimately, while this study demonstrates the potential of machine learning in analysing clinical data, additional research is required to validate these results for use in real-world clinical settings.

## Conclusion

Predictive diagnostic approaches to identifying high-risk patients leverage advanced deep learning (DL) techniques and data-driven models. By analysing clinical and demographic data, this strategy proves highly effective in facilitating the early detection of at-risk patients while enhancing the accuracy and effectiveness of interventions to improve patient outcomes. All these methods provide the expenses related to the laboratory parameter-driven approach, which ultimately supports patient-specific risk assessments and comprehensive population health management. Most laboratory parameters available in the market can be employed to create helpful tools, such as laying out the framework. Therefore, these fixed parameters have a significant impact on improving healthcare delivery and diagnostic accuracy. The technique was able to reliably and accurately stratify patients based on accuracy 83%, precision 80%, recall 78%, and F1 score 78%. The XGBoost + Optuna model has enhanced performance compared to other modern diagnostic strategies. It still presents critical limitations to clinical application. The F1 score of 78% indicates that, despite the model’s overall effectiveness, approximately 22% of actual high-risk patients were not correctly identified, resulting in 14 false negatives, as per the matrix confusion. This rate of false negatives is particularly concerning in a clinical context, where failure to detect high-risk individuals could lead to adverse outcomes.

The presence of these false negatives, as reflected by the recall value and confusion matrix, highlights a significant limitation that must be addressed before real-world deployment. Several factors may contribute to these misclassifications, including the quality of input data, lack of population diversity in training datasets, and constraints in real-time implementation. These challenges impact both the model’s performance and its generalizability across heterogeneous patient populations. Therefore, further research and validation studies are necessary to ensure the model’s reliability and safety in diverse clinical environments.

The proposed diagnostic strategy is influenced by the availability and quality of laboratory data, which may vary from one healthcare setting to another. However, one of the limitations of this study is the lack of external validation and the absence of a direct comparison with standard clinical diagnostic tools, such as the Oral Glucose Tolerance Test (OGTT). While our model demonstrated enhanced performance metrics, benchmarking against established clinical practices is essential to assess its potential role in real-world diagnostics. Future studies should focus on validating the model across diverse populations and settings, and on directly comparing its diagnostic utility with traditional methods to ensure clinical reliability and acceptance.

Future research can integrate Electronic Health Record (EHR) data for real-time predictions, utilise both structured and unstructured data, and add longitudinal data to account for patient changes over time. This highlights the current lack of external validation and underscores the need for broader testing across varied populations and clinical contexts to ensure the model’s applicability and robustness outside the study’s specific dataset.

## Data Availability

The datasets generated and/or analyzed during the current study are available in the Kaggle repository, (https://www.kaggle.com/datasets/mathchi/diabetes-data-set).
